# *In vivo* evidence of pathogenicity of VPS35 mutations in the *Drosophila*

**DOI:** 10.1186/s13041-014-0073-y

**Published:** 2014-10-08

**Authors:** Hua-shan Wang, Joanne Toh, Patrick Ho, Murni Tio, Yi Zhao, Eng-King Tan

**Affiliations:** National Neuroscience Institute, 11 Jalan Tan Tock Seng, 308433 Singapore, Singapore; Department of Clinical Research, Singapore General Hospital, 169856 Singapore, Singapore; Department of Neurology, Singapore General Hospital, 169856 Singapore, Singapore; Duke-NUS Graduate Medical School, 8 College Road, 169857 Singapore, Singapore

**Keywords:** Parkinson’s disease, Drosophila, VPS35, Retromer complex

## Abstract

Mutations of VPS35, a component of the retromer complex have been associated with late onset familial Parkinson’s disease. The D620N mutation in VPS35 appears to be most prevalent, however, P316S was found in two cases within the same family and a control, whereas L774M was identified in 6 cases and 1 control. In vivo evidence of their pathogenicity is lacking. Here we investigated the *in vivo* effects of P316S, D620N and L774M using *Drosophila* as a model. We generated transgenic human VPS35-expressing mutations and demonstrated that VPS35 D620N transgenic flies led to late-onset loss of TH-positive DA neurons, poor mobility, shortened lifespans and increased sensitivity to rotenone, a PD-linked environmental toxin, with some of these phenotypes observed for P316S but not in L774M transgenic flies. We conclude that D620N and to a smaller extent P316S are associated with pathogenicity in PD.

**Electronic supplementary material:**

The online version of this article (doi:10.1186/s13041-014-0073-y) contains supplementary material, which is available to authorized users.

## Introduction

Parkinson’s disease (PD) is a neurodegenerative disorder that affects 1% of the aging population. It is characterized by the loss of midbrain dopaminergic (DA) neurons in the substantia nigra region as well as accumulation of Lewy body aggregates. Although PD has been studied extensively, the molecular etiology is still unknown, with many trying to unravel the genetic determinants of PD [1]. VPS35 encodes for a subunit of the retromer, a coat-like protein complex that is involved in the recycling of membrane proteins. The retromer complex helps to guide protein sorting from the endosome-lysosome degradation pathway retrogradely to the Golgi apparatus and VPS35 functions as a platform for binding to other subunits (VPS26 and VPS29) with its distal ends in retromer assembly [2,3].

Recently, a number of reports determined a link between mutations in VPS35 and familial late-onset PD [4,5]. Successive studies showed that D620N mutation in VPS35 is the most prevalent and found to be associated with autosomal dominant PD in Caucasian and Japanese families but not in the Han Chinese population [6-9]. There are reports on other single mutations such as L774M and P316S but their pathogenicity remains unclear. P316S was found in two cases within the same family however, with a control subject also identified with the same mutation, genetic evidence for the pathogenicity of P316S remains unconvincing. L774M was identified in 6 cases and 1 control, hence the inconclusive evidence [5,10].

In flies, VPS35 was found to be involved in a number of physiological functions. Knockdown of VPS35 led to the inhibition of scavenger receptor endocytosis and VPS35 mutation resulted in the overproliferation and excessive differentiation of haemocytes, suggesting that it might have a tumor suppressor function. Also, there is likely a conserved function of VPS35 playing a role in endocytic trafficking [11]. In addition, VPS35 was shown to act in the same pathway with Rab9 to control tracheal length by positively regulating the luminal deposition of Serp, a specific cargo for the retromer-dependent endocytic trafficking [12]. Macleod et al., demonstrated that overexpression of VPS35 rescues dopaminergic neuronal loss and reduced lifespan caused by expression of LRRK2 G2019S in dopaminergic neurons, showing interaction between VPS35 and LRRK2 [13].

To date, there is limited *in vivo* evidence of pathogenicity of VPS35 mutations reported in single patients. To address these gaps in knowledge, utilizing *Drosophila* as a model, we investigated the pathogenicity of three reported VPS35 mutations, P316S, D620N and L774M *in vivo*. We showed evidence that D620N is the most pathogenic mutation in VPS35 followed by P316S, and that these mutations in VPS35 are linked to PD.

## Materials and methods

Experiments carried out adhered to ethical standards of the Research Center with regards to non-human experiments.

### Fly stocks

The following flies were used in this study: *dopa decarboxylase (ddc)*-GAL4 and *yellow white (yw)* (Bloomington *Drosophila* Stock Center). Human VPS35-expressing flies were created by generating transgenic human VPS35 wild type or mutant (P316S, D620N and L774M) cDNA containing a Hemagglutinin (HA) tag at the C-terminus and inserted into the pUAST-attB plasmid, which will allow the UAS constructs to land into a chosen attP site in the fly genome during microinjection. Constructs were then sent for microinjection into *Drosophila* embryos (BestGene). Flies were raised on standard yeast-cornmeal-agar medium at 25°C with 12-hour light and dark cycle.

### Western blot, immunofluorescence and confocal microscopy

Protein was extracted from fly head homogenates and equal amounts of protein from the various genotypes were resolved by SDS-PAGE. HA-tagged VPS35 was detected using mouse anti-HA (1:1000, Santa Cruz). Flies were aged to day 20 and 60 after eclosion, before fly brains were dissected, fixed and stained according to published protocols [14]. Brains were probed with rabbit anti-tyrosine hydroxylase (1:500, Sigma-Aldrich) and mouse anti-HA (1:200, Santa Cruz).

### Climbing, lifespan assays and rotenone treatment

Climbing assay was performed as described previously [15]. To determine adult lifespan, 100 flies from each genotype under the direction of *ddc*-GAL4 were maintained on standard media. Newly eclosed adult flies were transferred into vials containing fresh media every 3 days and mortality was scored daily. Age-matched *ddc*-GAL4/+flies used as controls. In rotenone-treated flies, flies were fed with cornmeal-agar medium containing 500 μM rotenone (Sigma), which was first dissolved in DMSO, immediately after eclosion and during entire experimental period.

### Statistical analysis

Quantitative data are expressed as mean ± SEM, unless otherwise stated. Statistical significance for climbing assay and differences in the number of TH-positive DA neurons were analyzed using one-way Anova with Bonferroni’s post hoc test, unless otherwise stated. Lifespan assay was analyzed with Log-rank test.

## Results

### VSP35 D620N promotes DA neurodegeneration and loss of locomotor activity

To study the pathogenic effects of VPS35 mutants *in vivo*, we generated the following HA tagged transgenic flies: wild type VPS35 as control, and mutant VPS35 flies, P316S, D620N and L774M. They were verified via immunostaining and immunoblot that the VPS35 from the different variants were expressed and in comparable amounts (Additional file 1: Figure S1).

**Figure 1:**
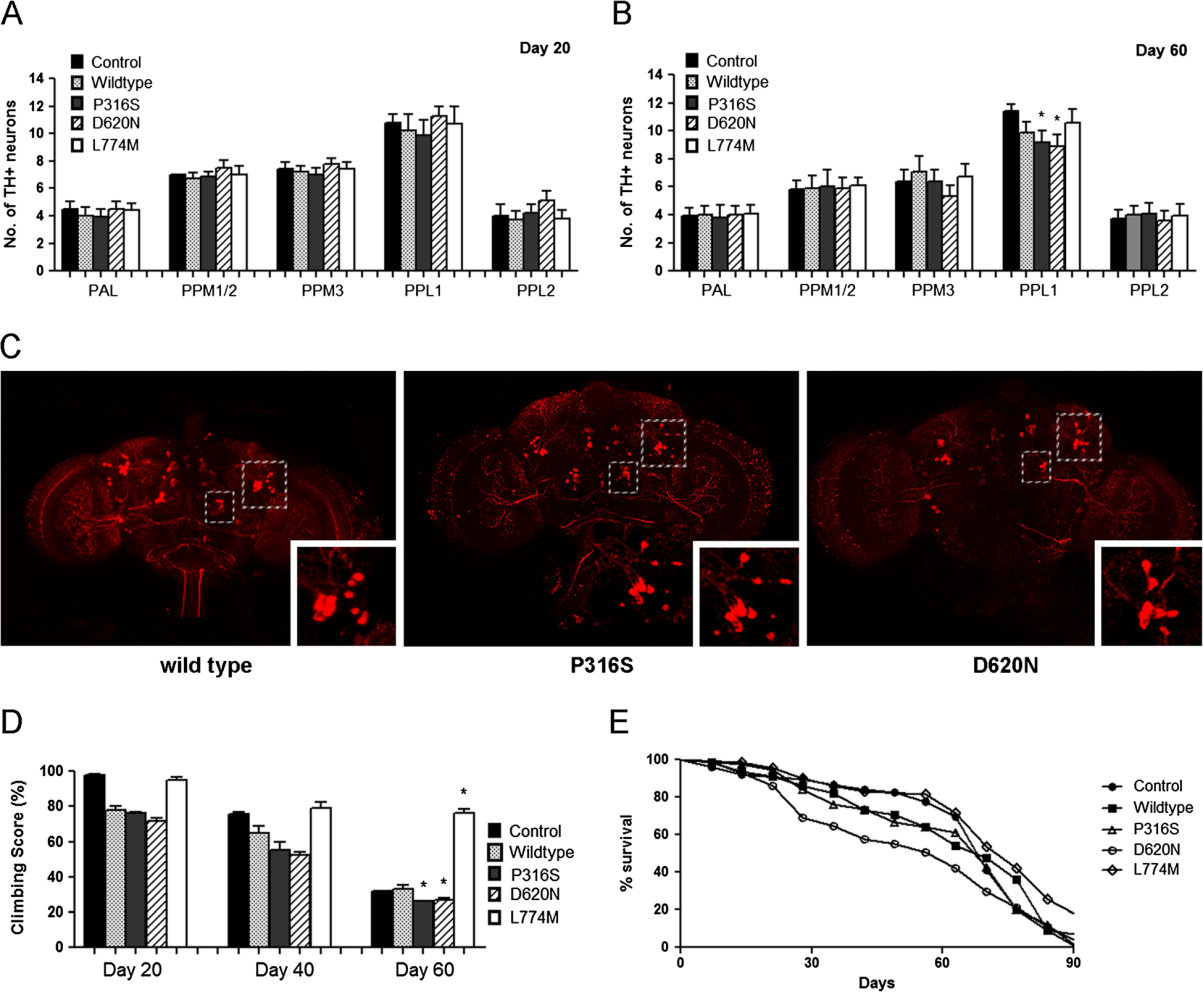
**Expression of VPS35 pathogenic variant D620N in flies result in DA neuronal degeneration and locomotive dysfunction. (A, B)** Bar graphs show number of TH-positive DA neurons in flies at 20 and 60 days after eclosion (n = 3, cohort of 10). **(C)** Representative confocal images of whole mount brains 60 days after eclosion of VPS35 wild type, P316S and D620N flies with magnified views of PPL1 cluster (boxed on the right). **(D)** Bar graph shows age-dependent climbing scores of female flies at different days after eclosion. Percentage of flies that reached the top of the column after 1 min were counted (n = 3, cohort of 20). **(E)** Survival curves were plotted as percentage of living flies. (n = 3, cohort of 100).

*ddc*-GAL4 flies, commonly used in *drosophila* PD research was used here for the overexpression of the various VPS35 transgenes in TH-positive DA neurons and to examine the gain-of-function effects of mutant VPS35 [16]. We first studied if DA neuronal integrity was affected, since this is a characteristic of PD patients, by comparing the number of TH-positive DA neurons (six clusters in each brain hemisphere). There was no apparent difference at 20 days after eclosion across all genotypes (Figure 1A) but in aged 60 days old flies, we saw a significant loss of TH-positive DA neurons in the PPL1 clusters of VPS35 D620N mutant flies (*p* < 0.05) compared to age matched wild type and control (*ddc*-GAL4/+) flies (Figure 1B, C). Interestingly, there was also significant difference between PPL1 clusters of control and wild type VPS35 flies (*p* < 0.05) (Figure 1B).

As DA neuronal loss has been linked to locomotor dysfunction, we compared the mobility of the various genotypes. At 20 and 40 days post eclosion, we observed a significant difference in climbing ability when comparing control flies to wildtype, P316S and D620N flies (*p* < 0.05) (Figure 1D). At 60 days post eclosion, there was a marked reduction in climbing abilities for all genotypes except L774M transgenic flies, with the climbing scores for VPS35 P316S and D620N flies significantly lower (*p* < 0.05) compared to age matched control and VPS35 wild type flies (Figure 1D). This correlated with the significant loss of DA neurons in VPS35 D620N mutant flies at 60 days. Interestingly, L774M transgenic flies displayed robust climbing even after 60 days of aging. There was a significant decrease (*p* < 0.05) in the median lifespan of D620N flies (63 days) compared to wild type and control flies (70 days) (Figure 1E).

### Rotenone treatment further exacerbated the pathogenicity of D620N VPS35 variant in DA neuron degeneration

**Figure 2:**
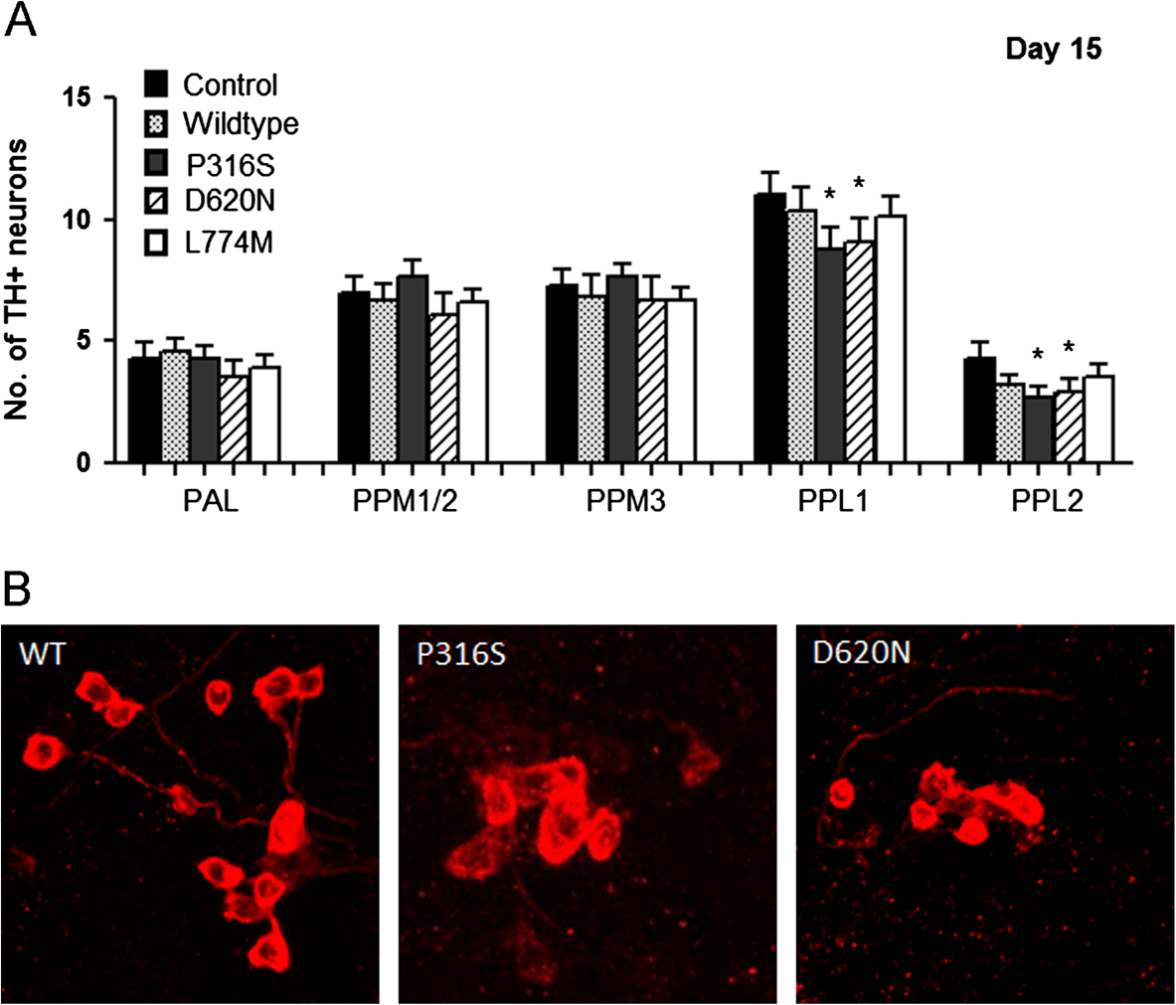
**Exposure of VPS35 P316S and D620N mutant flies to rotenone accelerates DA neuronal loss. (A)** Bar graph shows the quantification of the number of DA neurons in different clusters of various fly species 15 days after rotenone treatment (n = 3, cohort of 10). **(B)** Representative confocal images showing TH-positive DA neurons in the PPL1 cluster of rotenone treated fly species in VPS35 wild type, P316S and D620N flies.

We challenged the various transgenic VPS35-expressing flies with rotenone to examine their relative susceptibility of their TH-positive DA neurons to degeneration in response to a PD-linked environmental toxin. We observed a faster loss (15 days) of DA neurons in the PPL1 clusters of rotenone-treated flies expressing VPS35 P316S or D620N flies compared to age-matched control flies. Accelerated loss of TH-positive DA neurons was also observed in the PPL2 cluster of VPS35 P316S and D620N flies (*p <* 0.05) (Figure 2). Again, we did not find any loss of TH-positive DA neurons in flies expressing VPS35 L774M as rotenone treatment failed to enhance any loss of TH-positive DA neurons. These results suggest that D620N VPS35 flies are highly susceptible to rotenone DA neurodegeneration.

## Discussion

VPS35 is a component of the retromer complex recently linked to autosomal dominant late onset PD and *in vivo* evidence of pathogenicity of VPS35 mutations in single patient is lacking. Here, we characterized three reported variants of VPS35, P316S, D620N and L774M, in flies and provided *in vivo* evidence of the pathogenicity of VPS35 D620N mutation. We demonstrated that the expression of VPS35 D620N and to a smaller extent P316S, lead to the loss of TH-positive DA neurons, locomotor dysfunction and reduced lifespan.

The C-terminal amino acid residues 307 – 796 form an α-solenoid fold that fits into the metal-binding site of VPS29, suggesting that the known mutations of VPS35 might interfere with the interaction and assembly with VPS29, and disrupt retromer function in trafficking proteins intracellularly [17,18]. D620N is at the region where VPS35 binds not only to VPS29, but also accessory proteins such as cation-independent mannose-6-phosphate receptor (CI-MPR) and SNX1, whereby the former is essential for efficient anterograde transport of soluble hydrolases and also retrieval of mislocalized resident Golgi proteins, and the latter implicated in sorting of activated signaling receptors for lysosomal degradation [19]. Zavodszky et al. found that unlike a complete knockdown of VPS35 that eliminates retromer function, the D620N mutation affects retromer function linked to the WASH complex. They recently published findings showing that D620N mutation in VPS35 destabilizes the retromer-WASH complex interaction, resulting in reduced endosomal localization of the WASH complex, and that cells expressing the D620N VPS35 mutation exhibited defects in autophagy [20]. Defects in autophagy could be a contributor to PD pathogenesis as it may aggravate pathologies associated with PD, such as mitochondrial abnormalities, protein aggregation, increased reactive oxygen species and higher sensitivity to cell death. These may have contributed to the more severe phenotype observed in D620N flies. Although the precise mechanism of retromer dysfunction in PD is unclear, one report observed that the expression of VPS35 D620N led to endosomal alterations and defects in trafficking, which may partially explain its action in PD [21].

An interesting observation was the robust climbing of L774M mutation that appeared to have a protective effect during the climbing assay after flies were aged. As the mutation is at the C-terminus of VPS35 and is part of the binding region of VPS35 to VPS29, the mutation may have modulated structural changes in VPS35 such that it binds to VPS29 more efficiently. Another interesting observation was the toxic effect of over-expressing wild type VPS35, as we observed significant differences in the number of TH-positive neurons between age matched control and wild type flies as well as climbing abilities of age matched control and wild type flies at 20 and 40 days old. These results suggest that over-expressing wild type VPS35 alone causes the loss of DA neurons, and impairs locomotion even though Macleod and colleagues observed that the over-expression of wild type VPS35 rescued DA neuronal loss and reduced life span caused by LRRK2 G2019S expression in DA neurons [13]. In addition, the over-expression of D620N and P316S mutations aggravate this effect, which is consistent with the dominant nature of these mutations.

In a recent study by Tsika and colleagues, the common VPS35 D620N mutation did not compromise the protein stability, localization to endosomal and lysosomal vesicles, or the vesicular sorting of retromer cargo in rodent primary neurons or patient-derived human fibroblasts, suggesting that there is no loss of function. Instead, the overexpression of D620N VPS35 induced marked degeneration of substantia nigra dopaminergic neurons and axonal pathology in a rat model, implying that dominant VPS35 mutations lead to neurodegeneration in PD, which is similar to our results in flies [22].

We observed that the pathogenic process caused by single mutations of D620N and P316S can be exacerbated by rotenone, an environmental mitochondrial complex I inhibitor that has been linked to PD. Other findings have shown that the retromer complex regulates the transport of mitochondrial-anchored protein ligase to peroxisomes and that enhanced VPS35 expression protects dopaminergic cells against mitochondrial stress, suggesting that there might be a connection between VPS35 and pathogenesis of PD via mitochondrial stress [23,24].

Our study in characterizing the various VPS35 mutations will contribute to the understanding of VPS35-induced PD, with *in vivo* confirmation of the pathogenicity VPS35 D620N mutation. Further functional and genetic studies can be carried out to resolve the underlying mechanisms of VPS35 in PD, in addition to studying the interactions between VPS35 and other PD associated genes, which might provide important insights in the identification of more effective drug or molecular therapeutic targets for PD patients with VPS35 mutations.

## Additional file

Additional file 1: Figure S1. HA-tagged VPS35 transgenes tested for expression and protein levels. **(A)** Immunostaining of whole-mount adult fly brains expressing wild type or mutant human VPS35 in TH + neurons by overexpressing HA-tagged VPS35 variants in TH + neurons using *ddc*-GAL4. Top row: anti-HA. Middle row: anti-TH. Bottom row: Merged images of anti-HA (green) and anti-TH (red). **(B)** An anti-HA immunoblot of adult brain lysates prepared from control (*elav*-GAL4/+) or transgenic flies expressing the various VPS35 variants (*elav*-GAL4 > VPS35 variants).
