# Supplementary figures and images for: In vivo evidence of pathogenicity of VPS35 mutations in the Drosophila
Source: Mol Brain. 2014 Oct 8;7:73. doi: 10.1186/s13041-014-0073-y (PMC4193144; doi:10.1186/s13041-014-0073-y)

**
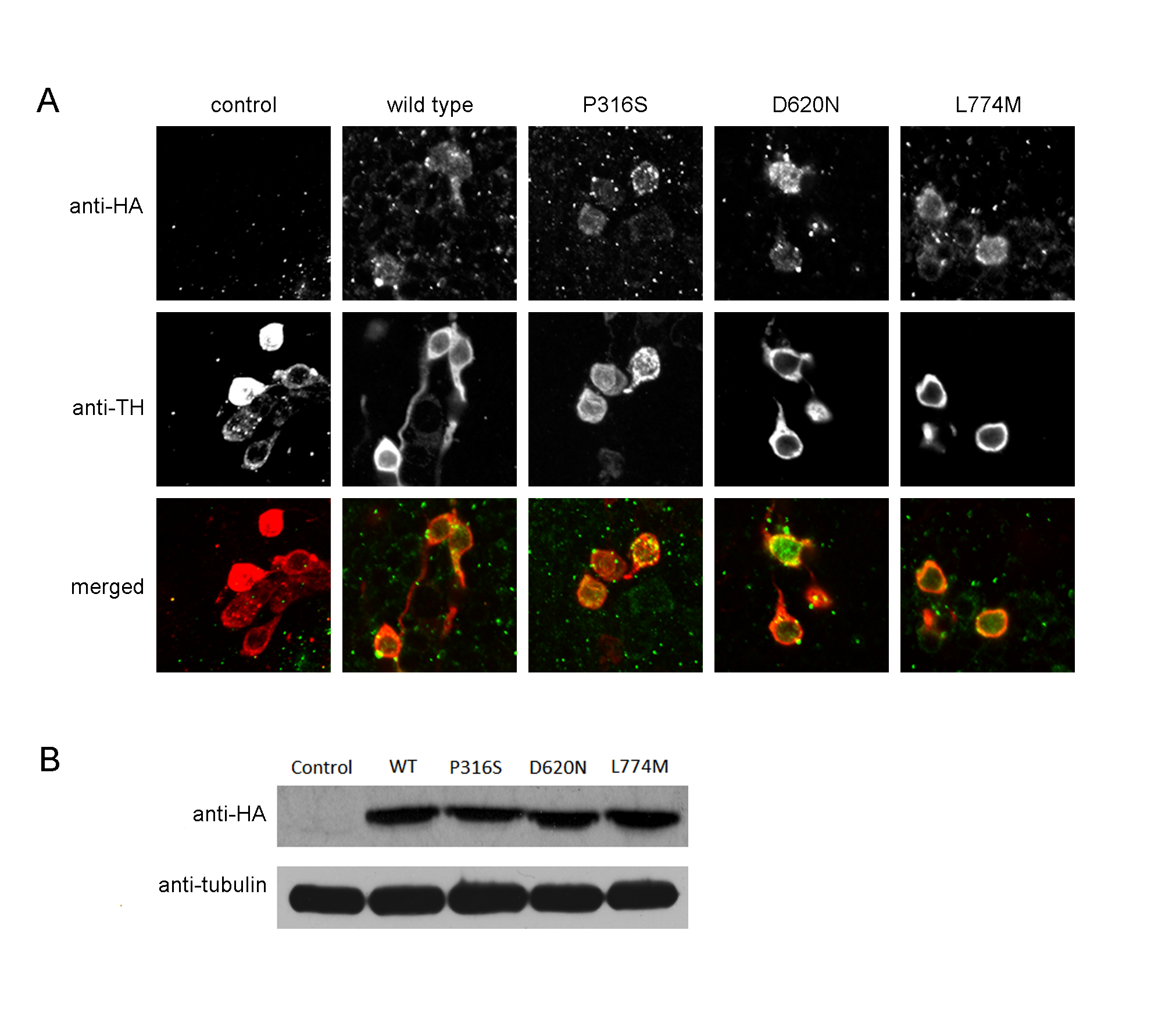
**

Supplement: Additional file 1: Figure S1. — HA-tagged VPS35 transgenes tested for expression and protein levels. (A) Immunostaining of whole-mount adult fly brains expressing wild type or mutant human VPS35 in TH + neurons by overexpressing HA-tagged VPS35 variants in TH + neurons using ddc-GAL4. Top row: anti-HA. Middle row: anti-TH. Bottom row: Merged images of anti-HA (green) and anti-TH (red). (B) An anti-HA immunoblot of adult brain lysates prepared from control (elav-GAL4/+) or transgenic flies expressing the various VPS35 variants (elav-GAL4 > VPS35 variants). [file 13041_2014_73_MOESM1_ESM.docx]
